# Substance use behavior and its lifestyle-related risk factors in Bangladeshi high school-going adolescents: An exploratory study

**DOI:** 10.1371/journal.pone.0254926

**Published:** 2021-07-21

**Authors:** Mst. Sabrina Moonajilin, Md Khalid Ibne Kamal, Firoj al Mamun, Mariam Binte Safiq, Ismail Hosen, Md. Dilshad Manzar, Mohammed A. Mamun

**Affiliations:** 1 Department of Public Health and Informatics, Jahangirnagar University, Savar, Dhaka, Bangladesh; 2 CHINTA Research Bangladesh, Savar, Dhaka, Bangladesh; 3 Department of Nursing, College of Applied Medical Sciences, Majmaah University, Al Majma’ah, Saudi Arabia; Shahjalal University of Science and Technology, BANGLADESH

## Abstract

Substance abuse is a major concern worldwide and is increasing rapidly in Bangladesh. However, there are no prior studies concerning lifestyle-related factors that influence adolescents’ substance use behavior. Therefore, the present study investigated the prevalence of substance use and its associated sociodemographic and lifestyle-related risk factors among a total of 424 Bangladeshi high school-going adolescents through a structured questionnaire interview study. The survey questionnaire consisted of socio-demographics, lifestyle-related information, and substance use-related questions. For data analysis, descriptive and inferential statistics were performed using SPSS (Statistical Package for Social Science) version 22.0, and a *p*-value of <0.05 determined statistical significance. Results showed that 21.2%, 14.4%, and 15.1% of the participants reported smoking, using a drug, and consuming alcohol, respectively, at least once during their lifespan; whereas the current (i.e., past-month) rates were reported to be 10.4%, 2.8%, and 3.1%, respectively. Overall, the current substance use risk factors were identified as being male, not being from science academic background, having less family influence on personal life, irregular teeth brushing, being smartphone users, using a smartphone for a longer time, and being late-night sleepers. From the list of identified risk factors of substance use, those that are modifiable may be targeted to evolve a prevention program to manage this problem in Bangladeshi adolescents.

## 1 Introduction

Substance abuse among adolescents is a substantial public health issue throughout the world. Recently, the World Health Organization [1] estimated that more than 275 million people use illicit drugs, accounting for 5.6% of the global population, and 31 million drug users have an addiction. Adolescence is considered to be the most transitionary period for one’s life dealing with sudden physiological and psychosocial changes. The changes may involve various unhealthy lifestyles and behaviors (e.g., smoking, alcohol consumption, illegal drug use) in many adolescents [2]. Substance abuse is the hazardous or harmful use of psychoactive substances (e.g., tobacco, alcohol, illicit drugs) [2–4]. The first onset of substance abuse is usually reported during adolescence [5]; more than 90% of addicted people with substance abuse behavior are introduced to substances before starting their adulthood [6].

Substance abuse often results in dependence syndrome, which includes: difficulties in controlling drug use; strong desire for taking a drug; notwithstanding negative repercussions, continuing to utilize it; drug usage is given a higher importance than other activities, and on rare occasions a physical retreat state [3]. Substance use has negative consequences on developmental and transitional years and affects the users throughout adulthood [2]. The adverse effects of substance use may express at various levels: individuals, their families, and communities. The effects of these problems have various dimensions, such as mental health problems, physical health, and economic consequences [2,5,7]. Adolescent drug abuse is associated with depression, sleep problems, anxiety, low self-esteem, aggressiveness, anti-social behavior, delinquency, crime, and rebelliousness [8–11]. Smoking initiation in early adolescence substantially increases future nicotine dependency risk [12], whereas alcohol consumption and tobacco use during this developmental stage may lead to experimentation with more serious addictive substances (i.e., heroin, hashish, narcotics) later on [13].

According to the Department of Narcotics Control (of Bangladesh), drug addiction is increasing dramatically in the country. For instance, 114 patients were treated daily in the year 2019, amounting to 104 and 69 people for 2018 and 2017, respectively [14]. Approximately 2.5 million Bangladeshis are addicted to drugs, and 80% are adolescents and young people of 15–30 years [15]. Therefore, there is an increasing tendency of substance use in Bangladesh, and the problem is more concerning among adolescents. However, there is a need to investigate current trends as most of the available studies published findings based on the survey reports that were carried out 10–15 years ago [16–20]. No prior studies concerning adolescent substance use and its risk factors have been carried out in the country. Information regarding substance use risk factors is essential in preventing these behaviors by incorporating risk factor-related information into policy, parental role, and school environment [21]. Hence, the present exploratory study investigates substance use behavior and its association with sociodemographic and lifestyle-related factors in a Bangladeshi high school-going adolescent sample.

## 2 Methods

### 2.1 Study site and participants

The interview administrators conducted a cross-sectional interview study across three randomly selected high schools in the Savar area, Dhaka, Bangladesh, from July 2017 to April 2018. In Bangladesh, the higher secondary school consists of two years of schooling (i.e., grade XI and grade XII), and one grade was randomly selected. This study utilized a 3-stage cluster sampling technique, where in the first stage, the schools were selected based on the proportion of the number of students enrolled. At the second stage, students’ grade was chosen randomly, and at the final stage, students age was selected. However, data was collected among all those students who were present in school on the survey day. In brief, the inclusion criteria were: (i) XI grade students, (ii) aged between 17–19 years, and (iii) present in the school during the survey time. Besides, the students were able to withdraw their participation during any stage of the interview. Around 450 students (approximately 150 students from each institute) were eligible for the interviews, and 432 partook in the interviews. After the removal of incomplete survey questionnaires, a total of 424 participants were included in the final analysis. The research questionnaire was structured in its form, and it was developed in Bangla, the native language of the participants, and later on translated to English for further analysis (in the S1 File, the English version questionnaire is provided). The questionnaire included items related to sociodemographic, lifestyle, and substance use behaviors.

### 2.2 Measures

#### 2.2.1. Sociodemographic information

These sociodemographic parameters were recorded: gender, study group, religion, performing exercise daily or playing daily, residing place during early ten years of life, current place of residence-living with their own family or not. Moreover, the presence/absence of parental influence on the adolescents was recorded. This information assessed if school-going Bangladeshi adolescents were at their liberty to decide what to choose for their education and other aspects of life.

#### 2.2.2. Lifestyle-related information

Lifestyle-related questions were based on: (i) hygiene practice, (ii) food habit, (iii) technology use behavior, (iv) sleep behavior, and (v) self-medication practice. Hygiene practice included items on brushing teeth, wearing clean clothes, washing hands, and using soap during bath. Whereas food habits were assessed by having daily breakfast, maintaining a balanced diet, etc. Factors related to technology use collected information on personal electronic device use and the daily duration of smartphone use. Sleep-related items recorded information about bedtime, sleep latent time, and total sleep time. Lastly, the practice of self-medication was assessed in the study.

#### 2.2.3. Substance abuse behaviors

Following previous studies, the habit of tobacco, alcohol, and illicit drugs was recorded to assess use of different types of substances [2,4]. For each of these substances, the participants were asked two questions: (i) if they had ever used any type of these substances, and (ii) if they had used any of these substances in the past month. The current substance use status was assessed based on the participants’ substance use in the past month [16,18,22]. If the participants used any of the aforementioned substances (i.e., tobacco, alcohol, and drug), they were referred to as substance users. Substance use was the dependent variable in this study, which had a dichotomous response (either “yes” or “no,”). Lastly, as smoking is common in Bangladesh, a question was included assessing the frequency of daily smoking.

### 2.3 Ethical considerations

The protocol was reviewed and approved by the Department of Public Health and Informatics, Jahangirnagar University, Savar, Dhaka, Bangladesh. Permission from each school principal was taken before implementing the study. Before conducting the interview, each student was informed about the purpose of the study, and informed consent was obtained. Subject data confidentiality and anonymity were maintained throughout the study.

### 2.4 Statistical analysis

The completed questionnaire was collected and checked for completeness and clarity of information. The data from the completed questionnaire was analyzed by SPSS software (Version 22.0). Bar charts were generated to illustrate descriptive statistics by Microsoft Excel. Descriptive statistics were presented with frequency tables. The association was illustrated with the Chi-square test and binary logistic regression considering the current substance use as the dependent variable. A *p*-value <0.05 was considered statistically significant, and odds ratios (OR) with 95% confidence intervals were estimated.

## 3 Results

### 3.1 Characteristics of the participants

In **Table 1**, the distribution of sociodemographic characteristics is presented. Out of a total of 424 participants, the majority were male (53.5%, n = 227), from a science background (56.1%), most of the participating adolescents were Muslims (93.2%), lived with family (91.5%), had their personal living room in the house (76.2%) and came from Upazilla town (53.8%). Lifestyle-related information is presented in **Table 2**. Among the respondents, 52.6% brushed their teeth regularly, 95% had the habit of wearing clean clothes. The majority reported completing their breakfast in the morning regularly (61.8%), 57.3% consumed fast food, and 73.8% reported maintaining a balanced diet. About 72.2% of students owned a smartphone, and 20.3% reported using it for more than 4 hours daily. Lastly, 21.2% slept less than 6 hours daily, and 57.1% reported practicing self-medication.

**Table 1:** Distribution of the sociodemographic variables across gender.

Variables	Total (n; %)	Male (*n;* %)	Female (*n;* %)	χ^2^ test value	df	*p*-value
**Gender**						
Male	227; 53.5%	-	-	-	-	-
Female	197; 46.1%	-	-			
**Study group**						
Science	238; 56.1%	128; 56.4%	110; 55.8%	2.892	2	0.236
Business	133; 31.4%	76; 33.5%	57; 28.9%			
Arts	53; 12.5%	23; 10.1%	30; 15.2%			
**Religion**						
Muslim	395; 93.2%	211; 93.0%	184; 93.4%	0.33	1	0.855
Others	29; 6.8%	16; 7.0%	13; 6.6%			
**Daily exercise or play**						
Yes	148; 34.9%	103; 46.2%	45; 23.4%	23.274	1	**<0.001**
No	267; 63.0%	120; 53.8%	147; 76.6%			
**First ten years of life, lived in**						
Metropolitan city	28; 6.6%	15; 6.6%	13; 6.7%	6.856	3	0.077
District town	56; 13.2%	34; 15.0%	22; 11.3%			
Upazilla town	228; 53.8%	110; 48.7%	118; 60.8%			
Village	108; 25.5%	67; 29.6%	41; 21.1%			
**Living with family**						
Yes	388; 91.5%	200; 88.9%	188; 95.4%	6.069	1	**0.014**
No	34; 8.0%	25; 11.1%	9; 4.6%			
**Having a personal room in the house**						
Yes	323; 76.2%	182; 81.6%	141; 73.1%	4.365	1	**0.037**
No	93; 21.9%	41; 18.4%	52; 26.9%			
**Having a good family influence on life (perception)**						
Yes	385; 90.8%	201; 91.4%	184; 96.3%	4.264	1	**0.039**
No	26; 6.1%	19; 8.6%	7; 3.7%			

**Table 2:** Distribution of the lifestyle-related variables across gender.

Variables	Total (n; %)	Male (*n;* %)	Female (*n;* %)	χ^2^ test value	df	*p*-value
**Lifestyle factors–Hygiene practice**						
**Habit of brushing teeth daily**						
Regular	223; 52.6%	88; 38.9%	135; 68.9%	37.758	1	**<0.001**
Irregular	199; 46.9%	138; 61.1%	61; 31.1%			
**Habit of wearing clean clothes**						
Yes	403; 95%	210; 92.9%	193; 98.5%	7.517	1	**0.006**
No	19; 4.5%	16; 7.1%	3; 1.5%			
**Habit of washing hand before taking meal**						
Yes	270; 63.7%	130; 58.6%	140; 71.1%	7.126	1	**0.008**
No	149; 35.1%	92; 41.4%	57; 28.9%			
**Habit of using soap during daily bath**						
Yes	384; 90.6%	197; 87.9%	187; 94.9%	6.366	1	**0.012**
No	37; 8.7%	27; 12.1%	10; 5.1%			
**Lifestyle factors–Food habit**						
**Habit of taking daily breakfast in the morning**						
Yes	262; 61.8%	151; 66.5%	111; 56.3%	4.625	1	**0.032**
No	162; 38.2%	76; 33.5%	86; 43.7%			
**Habit of eating fast food at restaurant**s						
Yes	243; 57.3%	135; 60.8%	108; 56.5%	0.772	1	0.380
No	170; 40.1%	87; 39.2%	83; 43.5%			
**Habit of taking balanced food**						
Yes	313; 73.8%	162; 84.4%	151; 85.3%	0.063	1	0.802
No	56; 13.2%	30; 15.6%	26; 14.7%			
**Amount of daily water intake (in average number of glasses)**						
5–6	236; 55.7%	107; 47.8%	129; 66.5%	15.384	2	**<0.001**
7–8	128; 30.2%	80; 35.7%	48; 24.7%			
9–10	54; 12.7%	37; 16.5%	17; 8.8%			
**Lifestyle factors–Technology use behavior**						
**Having an electronic device like a computer or television in a personal room**						
Yes	196; 46.2%	123; 58.6%	73; 42.0%	10.515	1	**0.001**
No	188; 44.3%	87; 41.4%	101; 58.0%			
**Having a personal smart-phone**						
Yes	306; 72.2%	181; 87.0%	125; 65.4%	25.926	1	**<0.001**
No	93; 21.9%	27; 13.0%	66; 34.6%			
**Average smartphone usage time**						
1–2 hours	140; 33.0%	56; 25.9%	84; 49.7%	27.831	3	**<0.001**
2–3 hours	95; 22.4%	56; 25.9%	39; 23.1%			
3–4 hours	64; 15.1%	40; 18.5%	24; 14.2%			
More than 4 hours	86; 20.3%	64; 29.6%	22; 13.0%			
**Lifestyle factors–Sleep behavior**						
**Habit of going to bed for sleep**						
Within 11pm	116; 27.4%	48; 21.5%	68; 34.5%	17.574	3	**0.001**
Within 12am	173; 40.8%	89; 39.9%	84; 42.6%			
Within 1am	86; 20.3%	52; 23.3%	34; 17.3%			
After 1am	45; 10.6%	34; 15.2%	11; 5.6%			
**Usual sleep latent time**						
Within 10–15 minutes	197; 46.5%	94; 42.5%	103; 52.6%	5.515	3	0.138
Within 15–30 minutes	114; 26.9%	62; 28.1%	52; 26.5%			
Within 30–45 minutes	56; 13.2%	33; 14.9%	23; 11.7%			
After 45 minutes	50; 11.8%	32; 14.5%	18; 9.2%			
**Duration of total sleep**						
4 hours or less	22; 5.2%	11; 4.9%	11; 5.6%	2.664	2	0.264
5 hours	63; 14.9%	28; 12.5%	35; 17.9%			
6 hours or more	334; 78.8%	185; 82.6%	149; 76.4%			
**Lifestyle factors–Self-medication practice**						
**Any medicine taken without a prescription**						
Yes	242; 57.1%	143; 63.8%	99; 50.3%	7.915	1	**0.005**
No	179; 42.2%	81; 36.2%	98; 49.7%			
**Type of medicine taken without a prescription**						
Pain killer	48; 11.3%	26; 16.1%	22; 16.9%	2.919	3	0.404
Medicine for fever	174; 41.0%	102; 63.4%	72; 55.4%			
Medicine for common gastric alimentsand Vitamins	63; 14.9%	31; 19.3%	32; 24.6%			
Medicine for sleep and others	6; 1.4%	2; 1.2%	4; 3.1%			

### 3.2 Gender-based distribution of substance use

**Fig 1:**
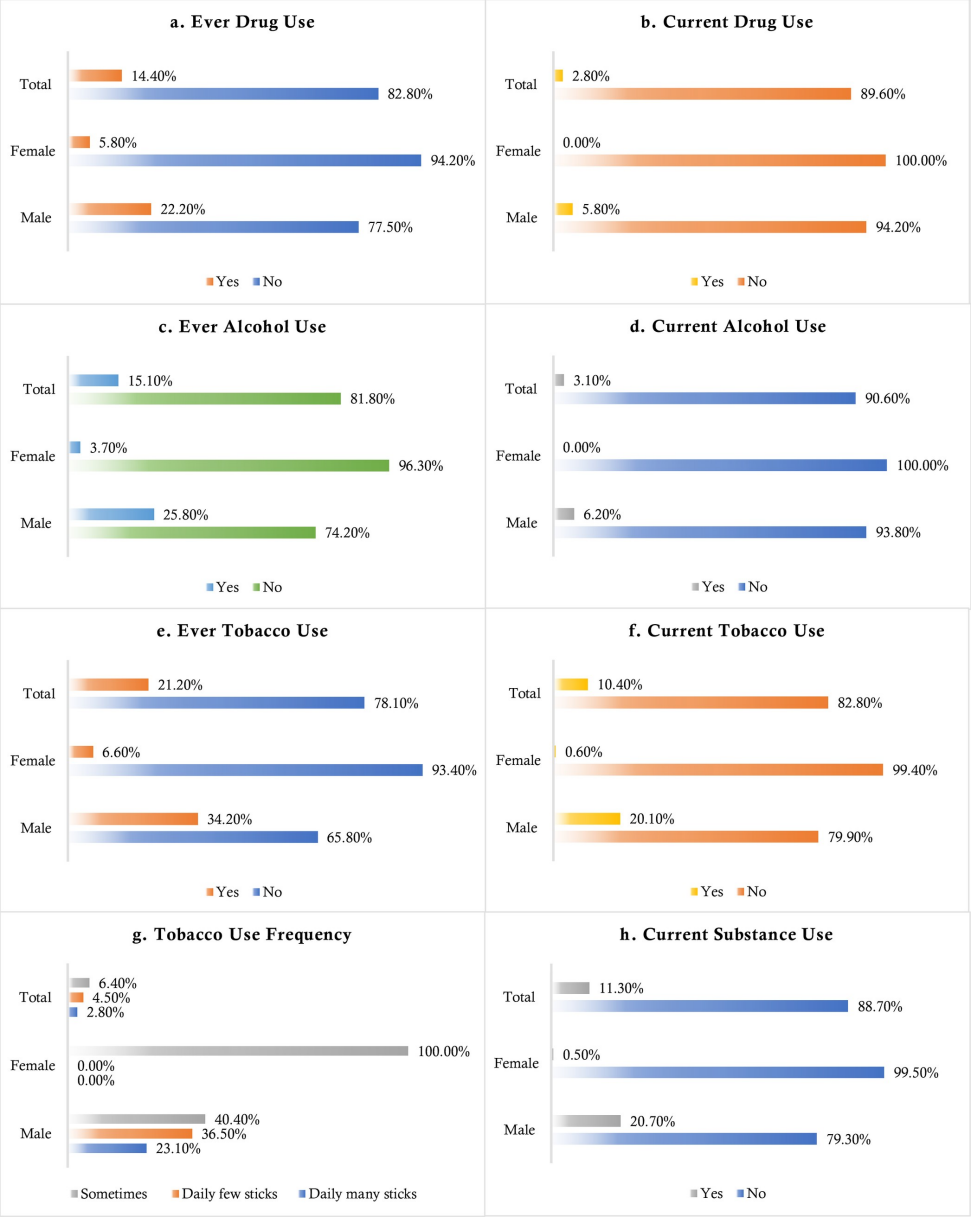
Distribution of the substance use behaviors across gender.

**Fig 1** shows the gender-based distributions of the substance use-related variables. Around 21.2% of students were ever involved with smoking, whereas it was 14.4% and 15.1% for drug use and alcohol consumption, respectively; the rates were statistically significant with gender-based distribution (χ^2^ = 47.439, *p*<0.001; χ^2^ = 22.726, *p*<0.001; and χ^2^ = 37.981, *p*<0.001, respectively). Similarly, current rates of smoking (10.4%), drug use (2.8%) and alcohol consumption (3.1%) were significantly associated with gender (χ^2^ = 37.829, *p*<0.001; χ^2^ = 10.951, *p*<0.001; and χ^2^ = 11.848, *p*<0.001 respectively). Male participants were more frequent smokers, whereas all of the females reported being a smoker at some point in time during their lives (χ^2^ = 7.684, *p* = 0.021). Finally, any type of current substance use was reported in 11.3% of students, which was also predominantly high in male participants (χ^2^ = 7.684, *p* = 0.021) (**Fig 1**).

### 3.3 Risk factors of substance use

**Tables 3 and 4** represent socio-demographic and lifestyle-related risk factors associated with substance use. Being male (OR = 51.178, CI = 6.989–374.772, *p*<0.001); not belonging to science academic background (OR = 0.263, CI = 0.117–0.591 [science], OR = 0.569, CI = 0.25–1.275 [business], reference = arts, *p* = 0.002); less family influence on personal life (*p* = 0.009); irregular teeth brushers (*p* = 0.006); not using soap during daily bath (*p* = 0.034); consuming fast food (OR = 2.216, CI = 1.114–4.406, *p* = 0.021); owning personal smartphone (OR = 7.439, CI = 1.767–31.325, *p*<0.001); more use of smartphone (*p*<0.001); and late-night sleepers (*p*<0.001) were significantly associated with substance use (**Tables 3 and 4**).

**Table 3:** Distribution of the socio-demographic variables with respect to any type of current substance use.

Variables	Substance users (*n;* %)	Substance non-users (*n;* %)	χ^2^ test value	df	*p*-value	OR; 95% CI
**Gender**						
Male	47; 20.7%	180; 79.3%	42.856	1	**<0.001**	51.178 (6.989–374.772)
Female	1; 0.5%	196; 99.5%				Reference
**Study group**						
Science	17; 7.1%	221; 92.9%	12.069	2	**0.002**	0.263 (0.117–0.591)
Business	19; 14.3%	114; 85.7%				0.569 (0.254–1.275)
Arts	12; 22.6%	41; 77.4%				Reference
**Religion**						
Muslim	45; 11.4%	350; 88.6%	0.030	1	0.864	1.114 (0.324–3.830)
Others	3; 10.3%	26; 89.7%				Reference
**Daily exercise or play**						
Yes	20; 13.5%	128; 86.5%	1.097	1	0.295	1.389 (0.750–2.573)
No	27; 10.1%	240; 89.9%				Reference
**First ten years of life lived in**						
Metropolitan city	5; 17.9%	23; 82.1%	1.367	3	0.713	1.739 (0.557–5.428)
District town	6; 10.7%	50; 89.3%				0.960 (0.340–2.710
Upazilla town	24; 10.5%	204; 89.5%				0.941 (0.452–1.961)
Village	12; 11.1%	96; 88.9%				Reference
**Living with family**						
Yes	45; 11.6%	343; 88.4%	1.032	1	0.310	2.099 (0.487–9.057)
No	2; 5.9%	32; 94.1%				Reference
**Having a personal room in the house**						
Yes	37; 11.5%	286; 88.5%	1.178	1	0.278	1.589 (0.684–3.693)
No	7; 7.5%	86; 92.5%				Reference
**Having a good family influence on life (perception)**						
Yes	39; 10.1%	346; 89.9%	6.910	1	**0.009**	0.306 (0.121–0.774)
No	7; 26.9%	19; 73.1%				Reference

**Table 4:** Distribution of the lifestyle-related variables with respect to any type of current substance use.

Variables	Substance users (*n;* %)	Substance non-users (*n;* %)	χ^2^ test value	df	*p*-value	OR; 95% CI
**Lifestyle factors–Hygiene practice**						
**Habit of daily teeth brushing**						
Regular	16; 7.2%	207; 92.8%	7.503	1	**0.006**	0.419 (0.222–0.792)
Irregular	31; 15.6%	168; 84.4%				Reference
**Habit of wearing clean clothes**						
Yes	45; 11.2%	358; 88.8%	0.385	1	0.535	0.670 (0.188–2.391)
No	3; 15.8%	16; 84.2%				Reference
**Habit of washing hand before taking a meal**						
Yes	28; 10.4%	242; 89.6%	0.287	1	0.592	0.842 (0.449–1.580)
No	18;12.1%	131; 87.9%				Reference
**Habit of using soap during daily bath**						
Yes	39; 10.2%	345; 89.8%	4.473	1	**0.034**	0.410 (0.175–0.959)
No	8; 21.6%	29; 78.4%				Reference
**Lifestyle factors–Food habit**						
**Habit of taking daily breakfast in the morning**						
Yes	33; 12.6%	229; 87.4%	1.110	1	0.292	1.412 (0.741–2.690)
No	15; 9.3%	147; 90.7%				Reference
**Habit of eating fast food at restaurants**						
Yes	35; 14.4%	208; 85.6%	5.350	1	**0.021**	2.216 (1.114–4.406)
No	12; 7.1%	158; 92.9%				Reference
**Habit of taking balanced food**						
Yes	30; 9.6%	283; 90.4%	0.448	1	0.504	0.742 (0.309–1.783)
No	7; 12.5%	49; 87.5%				Reference
**Amount of daily water intake (in average number of glasses)**						
5–6	21; 8.9%	215; 91.1%	4.367	2	0.113	0.430 (0.189–0.976)
7–8	16; 12.5%	112; 87.5%				0.629 (0.265–1.491)
9–10	10; 18.5%	44; 81.5%				Reference
**Lifestyle factors–Technology use behavior**						
**Having an electronic device like a computer or television in a personal room**						
Yes	27; 13.8%	169; 86.2%	2.675	1	0.102	1.717 (0.893–3.302)
No	16; 8.5%	172; 91.5%				Reference
**Having a personal smart-phone**						
Yes	43; 14.1%	263; 85.9%	10.097	1	**<0.001**	7.439 (1.767–31.325)
No	2; 2.2%	91; 97.8%				Reference
**Average smartphone usage time**						
1–2 hours	6; 4.3%	134; 95.7%	23.006	3	**<0.001**	0.130(0.050–0.337)
2–3 hours	10; 10.5%	85; 89.5%				0.342 (0.152–0.773)
3–4 hours	9; 14.1%	55; 85.9%				0.476 (0.202–1.120)
More than 4 hours	22; 25.6%	64; 74.4%				Reference
**Lifestyle factors–Sleep behavior**						
**Habit of going to bed for sleep**						
Within 11pm	7; 6.0%	109; 94.0%	18.164	3	**<0.001**	0.158 (0.058–0.430)
Within 12am	16; 9.2%	157; 90.8%				0.251 (0.110–0.572)
Within 1am	11; 12.8%	75; 87.2%				0.361 (0.146–0.891)
After 1am	13; 28.9%	32; 71.1%				Reference
**Usual sleep latent time**						
Within 10–15 minutes	14; 7.1	183; 92.9%	11.885	3	**0.008**	0.242 (0.104–0.565)
Within 15–30 minutes	13; 11.4%	101; 88.6%				0.408 (0.171–0.972)
Within 30–45 minutes	6; 10.7%	50; 89.3%				0.380 (0.131–1.104)
After 45 minutes	12; 24.0%	38; 76.0%				Reference
**Duration of total sleep**						
4 hours or less	5; 22.7%	17; 77.3%	5.628	2	0.060	2.162(0.756–6.180)
5 hours	3; 4.8%	60; 95.2%				0.368 (0.110–1.227)
6 hours or more	40; 12.0%	294; 88.0%				Reference
**Lifestyle factors–Self-medication practice**						
**Any medicine taken without a prescription**						
Yes	32; 13.2%	210; 86.8%	2.434	1	0.119	1.666 (0.873–3.180)
No	15; 8.4%	164; 91.6%				Reference
**Type of medicine taken without a prescription**						
Pain killer	10; 20.8%	38; 79.2%	5.852	3	0.119	1.316 (0.138–12.574)
Medicine for fever	15; 8.6%	159; 91.4%				0.472(0.052–4.306)
Medicine for common gastric ailments and Vitamins	9; 14.3%	54; 85.7%				0.833 (0.087–7.986)
Medicine for sleep and others	1; 16.7%	5; 83.3%				Reference

## 4 Discussion

The substance use rate is increasing among adolescents in South Asian countries, including Bangladesh. Adolescent students are the most common victims of substance abuse, which ultimately reduces their school attendance, educational achievement, and quality of life. A previous report found that the average age of addiction in the Bangladeshi population is 22 years, ranging from 15 to 30 years [23]. In Bangladesh, healthcare facilities claim that nearly 10% of the outpatients report addiction-related complications involving heroin, marijuana, and phensedyl [23].

This study found that 10.4% of students were currently tobacco smokers, 2.8% used drugs, and 3.1% consumed alcohol, whereas the rates were 21.2%, 14.4%, and 15.1%, respectively, for lifetime users. The prevalence of current substance use was 11.3%. Though there is a lack of studies assessing similar objectives reported herein, the present findings can be compared to available previous survey studies which were carried out 10–15 years ago in the country. Therefore, the conclusions of this study are more likely to reflect the present prevalence of substance use and its risk factors among Bangladeshi adolescents.

However, available Bangladeshi literature suggests some heterogeneity in the findings. For instance, the prevalence rate of 8.4% and 35.6% for current and ever-in-lifetime smoking, respectively, were reported in an adolescent cohort aged between 11 and 17 years by the Global Youth Tobacco Survey for Bangladesh conducted in 2007 [16]. The rate of cigarette smoking among current users reported in the Urban Health Survey in 2006 was 53.6%, which was higher in the participants residing in urban slum areas (59.8%) vs. 46.4% among non-slum dwellers [17]. Utilizing the same dataset, another study reported a 42.3% rate of smoking among current users of the young slum dwellers aged between 15 to 24 years [18]. However, assessing only male participants, another study reported the current smoking rate to be 60%; this was based on the Bangladesh Demographic and Health Survey 2007 [20]. Based on the same survey dataset, 3.4% of the respondents were reported taking illicit drugs, including ganja (cannabis), charas (purified resinous extract of cannabis), phensidyl, pethidine, heroin, morphine, and injectable drugs in a period of 3-months preceding the survey. In the same study, 83.8% of the drug users reported using one type of drug [20]. A prior dataset from the Bangladesh Demographic and Health Survey 2004 estimated the prevalence of last 3-month illicit drug use as 4%, wherein ganja (3%) was the most frequently used drug followed by phensidyl (0.8%), heroin (0.3%), and charas (0.3%) [19]. But the past-month illegal drug use rate was higher- 9.1% (i.e., 3.2% drug injections, 2.8% ganja, 1.6% tari [toddy or palm wine]) in slum youth residents [18].

Studies regarding substance use were also carried out globally. An Indian study, the neighboring country of Bangladesh, found the lifetime prevalence of 6.14% and 0.6% for illicit substance use; 8.60% and 11.04% for tobacco use; and 7.37%, and 5.23% for alcohol consumption among the adolescent belonging to rural and urban students, respectively [24]. Among the Omani college students aged 21.0 (±2.8) years old, 41.3% and 29.9% were reported misusing any substance, including tobacco and alcohol, and without tobacco or alcohol, respectively [25]. The same study also identified tobacco as the most commonly used substance which prevalence was 23.5%, followed by alcohol at 10.7% [25]. While considering the Nigerian high school-going adolescents (of 11 and 12 grades), the lifetime and current rate of any substance use were 17.3% and 11.7%, respectively [26]. In addition, 27.6%, 16.3%, and 13.0% of the Iraqi high school students aged 14–19 years reported smoking cigarettes over a lifetime, last year, and last month, respectively; while 23.8%, 3.7%, and 1.4% were the lifetime prevalence of waterpipe smoking, alcohol consumption, and use of unprescribed tranquilizers, respectively [27]. The same study also identified that 34.3% of the adolescents reported using any of the substances sometimes during their lifetime [27]. To this end, it can be noted that the diversity of substance use in a particular cohort (medical students, for instance) can vary across the cultures, used methodology, and other factors [28–30]. Therefore, cross-cultural studies are needed to provide a better understanding of substance use across countries.

The present findings report that male participants are at a 50-time higher risk of being substance users, which is not uncommon in this context. For example, a previous Bangladeshi study observes similar findings for smoking and gul (powdered tobacco, habitual use is common in South-east Asia) usage (42.2% and 2.2% vs. 2.3% and 1.5%, respectively), although chewing tobacco is higher in female participants (21.6% vs. 19.4%) [31]. Similarly, male participants used more substances than females in Nepal [28] and Ethiopia [30]. Students of arts and commerce backgrounds were reported to have higher substance use behaviors. This may be because of less academic pressure compared to students belonging to science backgrounds. There is a trend of enrolling less meritorious students in arts and commerce courses in the country. This group often has a lower burden of expectation from families, which probably increases substance abuse risk [32].

Prevalence of substance use may increase due to several factors such as higher availability of the substances [17], peer influence [13,33], more pocket money, being influenced by seeing other’s engagement [16], for fun or partying [28], and family negligence [32]. Adolescent substance use behavior is also influenced by the average grade level, truancy, and religious involvement [34]. However, a limited number of previous studies assessed the influence of lifestyle-related variables on substance use behaviors, which was considered in the present study. Irregular sleep and greater daytime sleepiness were observed to predict higher substance use behavior in American adolescents [8]. Similarly, the present study alludes to participants reporting late-night sleeping hours and higher sleep latency as risk factors of being substance users. Owning a smartphone increased substance use risk by seven-fold, whereas more frequent smartphone usage was also a risky behavior. It is well-established that engaging in more technology use leads people to be addicted to technology, which is the most common feature of psychiatric suffering [35,36]. And people with psychiatric sufferings tend to indulge in substance use to escape emotional instabilities and avoid painful states [37,38].

The present study is limited by its nature (cross-sectional) and participants belonging to one region (i.e., Savar) and only XI grade students. Thus, further studies utilizing more rigorous methodologies are warranted. Despite these limitations, the study provides an preliminary assessment on lifestyle-related substance use risk factors. The findings may facilitate further studies and policy reformulation.

## 5 Conclusions

Adolescent substance use has become a major public health problem in developing countries, including Bangladesh. Therefore, implementing different interventions focusing on adolescent modifiable lifestyle-related risky behaviors reported herein are highly suggested. In addition, these prevention programs may explore ways to reduce substance use by interventions tailored to modify these lifestyles and other related risk factors/behaviors.

## Supporting information

S1 File English version of the questionnaire. (DOCX)

S2 File Dataset of the manuscript. (SAV)
