# Supplementary material for: Substance use behavior and its lifestyle-related risk factors in Bangladeshi high school-going adolescents: An exploratory study
Source: PLoS One. 2021 Jul 21;16(7):e0254926. doi: 10.1371/journal.pone.0254926 (PMC8294555; doi:10.1371/journal.pone.0254926)
Supplement: S1 File — (DOCX) [file pone.0254926.s001.docx]

Substance abuse behaviors and its lifestyle-related risk factors in Bangladeshi high school-going adolescents: An exploratory study

**Socio-demographic information**

1. Gender

- Male
- Female

2. In which group you are studying?

- Science
- Business
- Arts

3. What is your religion?

- Muslim
- Others (Hindu, Buddha, Christian)

4. Do you take part in daily exercise or play?

- Yes
- No

5. Where did you live in your first ten years of life?

- Metropolitan city
- District town
- Upazilla town
- Village

6. Are you living with your family?

- Yes
- No

7. Do you have a personal room in the house?

- Yes
- No

8. Do you think, your family have influence on your life?

- Yes
- No

**Lifestyle related factors- Hygiene practice**

1. Do you brush your teeth regularly?

- Yes
- No

2. Do you wear clean clothes regularly?

- Yes
- No

3. Do you wash your hands regularly before taking meal?

- Yes
- No

4. Do you use soap regularly during bath?

- Yes
- No

**Lifestyle related factors- Food habit**

1. Do you have your breakfast regularly?

- Yes
- No

2. Do you go to restaurants to eat fast food?

- Yes
- No

3. Do you take balanced food (i.e., taking carbohydrate, protein and fats properly?

- Yes
- No

4. On average how many numbers of glass of water do you drink?

- 5-6
- 7-8
- 9-10

**Lifestyle related factors – Technology use behavior**

1. Do you have an electronic device like a computer or TV in a personal room?

- Yes
- No

2. Do you own a smartphone?

- Yes
- No

3. How many hours on average do you use smartphone?

- 1-2 hours
- 2-3 hours
- 3-4 hours
- More than 4 hours

**Lifestyle related factors- Sleep behavior**

1. When do you go to bed?

- Within 11pm
- Within 12 am
- Within 1 am
- After 1 am

2. What is your usual latent sleep time?

- Within 10-15 minutes
- Within 15-30 minutes
- Within 30-45 minutes
- After 45 minutes

3. What is your total duration of sleep?

- 4 hours or less
- 5 hours
- 6 hours or more

**Lifestyle related factors- Self-medication practice**

1. Do you take any medicine without prescription?

- Yes
- No

2. What kind of medicine do you take without medicine?

- Pain killer
- Medicine for fever
- Medicine for gastritis and vitamins
- Medicine for sleep and others

**Substance abuse behaviors**

1. Did you drink alcohol once in your lifetime?

- Yes
- No

2. Do you currently drinking alcohol?

- Yes
- No

3. Did you take illicit drug once in your lifetime?

- Yes
- No

4. Do you currently take illicit drug?

- Yes
- No

5. Did you take tobacco once in your lifetime?

- Yes
- No

6. Do you currently taking tobacco?

- Yes
- No

7. How frequently do you smoke?

- Daily many sticks
- Daily few sticks
- Sometimes
